# Inulin Supplementation Mitigates Gut Dysbiosis and Brain Impairment Induced by Mild Traumatic Brain Injury during Chronic Phase

**DOI:** 10.33696/immunology.4.132

**Published:** 2022

**Authors:** Lucille M. Yanckello, Brian Fanelli, Scott McCulloch, Xin Xing, McKenna Sun, Tyler C. Hammond, Rita Colwell, Zezong Gu, Aaron C. Ericsson, Ya-Hsuan Chang, Adam D. Bachstetter, Ai-Ling Lin

**Affiliations:** 1 Sanders Brown Center on Aging, University of Kentucky, Lexington, KY, United States of America; 2 Department of Pharmacology and Nutritional Sciences, University of Kentucky, Lexington, KY, United States of America; 3 CosmosID Inc., Rockville, MD, United States of America; 4 Metabolon Inc., Durham, NC, United States of America; 5 Department of Computer Science, University of Kentucky, Lexington, KY, United States of America; 6 Department of Neuroscience, University of Kentucky, Lexington, KY, United States of America; 7 Department of Pathology & Anatomical Sciences, University of Missouri, Columbia, MO, United States of America; 8 Harry S. Truman Memorial Veteran Hospital, Columbia, MO, United States of America; 9 Department of Veterinary Pathobiology, University of Missouri, Columbia, MO, United States of America; 10 Spinal Cord and Brain Injury Research Center, University of Kentucky, KY, United States of America; 11 Department of Radiology, University of Missouri, Columbia, MO, United States of America; 12 Institute for Data Science &Informatics, University of Missouri, Columbia, MO United States of America

**Keywords:** Traumatic brain injury, Inulin diet, gut microbiome, Short chain fatty acids, Cerebral blood flow, MRI, Machine learning

## Abstract

Mild traumatic brain injury (mTBI) has been shown to acutely alter the gut microbiome diversity and composition, known as dysbiosis, which can further exacerbate metabolic and vascular changes in the brain in both humans and rodents. However, it remains unknown how mTBI affects the gut microbiome in the chronic phase recovery (past one week post injury). It is also unknown if injury recovery can be improved by mitigating dysbiosis. The goal of the study is to fill the knowledge gap. First, we aim to understand how mTBI alters the gut microbiome through the chronic period of recovery (3 months post injury). In addition, as the gut microbiome can be modulated by diet, we also investigated if prebiotic inulin, a fermentable fiber that promotes growth of beneficial bacteria and metabolites, would mitigate dysbiosis, improve systemic metabolism, and protect brain structural and vascular integrity when administered after 3 months post closed head injury (CHI). We found that CHI given to male mice at 4 months of age induced gut dysbiosis which peaked at 1.5 months post injury, reduced cerebral blood flow (CBF) and altered brain white matter integrity. Interestingly, we also found that Sham mice had transient dysbiosis, which peaked 24 hours after injury and then normalized. After 8 weeks of inulin feeding, CHI mice had increased abundance of beneficial/anti-inflammatory bacteria, reduced abundance of pathogenic bacteria, enriched levels of short-chain fatty acids, and restored CBF in both hippocampi and left thalamus, compared to the CHI-control fed and Sham groups. Using machine learning, we further identified top bacterial species that separate Sham and CHI mice with and without the diet. Our results indicate that there is an injury- and time-dependent dysbiosis between CHI and Sham mice; inulin is effective to mitigate dysbiosis and improve brain injury recovery in the CHI mice. As there are currently no effective treatments for mTBI, the study may have profound implications for developing therapeutics or preventive interventions in the future.

## Introduction

Approximately 1.6–3.8 million people sustain a mild traumatic brain injury (mTBI) in the US annually [1]. This amounts to the hospitalization of 100–300 per 100,000 young adults [1,2]. The Centers for Disease Control and Prevention reports that around 5.3 million people live with a permanent disability after mTBI [3], and there are currently no known restorative therapies [4].

Reduced cerebral blood flow (CBF) [5,6] and impaired white matter integrity (WMI) [7] are typical phenotypes of mTBI. Recent studies further show that mTBI significantly alters the balance of microbial communities in the gut of rodents and humans, known as gut dysbiosis [8–10].The effects of neurotrauma radiate to the intestine and alter the composition and diversity of the gut microbiome. This results in reduced levels of putatively beneficial bacteria which in turn leads to the reduction of short-chain fatty acids (SCFAs), bacterial metabolites that play a key role in modulating metabolism, vascular response, and inflammation system-wide [11,12]. Dysbiosis has been shown to occur in the acute phase as early as one day post injury in rodent models [9] and humans [10]. Elucidating that TBI induces dysbiosis pinpoints the gut microbiome as a potential target to protect against the secondary injury cascade known to follow TBI [13]. However, gut microbiome composition past the acute phase of mTBI (1–7 days) has yet to be characterized. This leaves a pivotal gap in the knowledge pertaining to the long-term changes in gut microbial structure after the initial injury. Further, it is unknown whether interventions that can modulate the microbiome, such as diet, could mitigate injury effects in the chronic phase.

The purpose of the study is two-fold. First, with a longitudinal study design, we determined the long-term effects of CHI on the gut microbiome, CBF, and WMI. Second, we determined if prebiotic inulin could mitigate injury-induced dysbiosis and restore CBF and WMI by modulating gut microbiome from 3 months post injury (mpi) to 5 mpi. We chose inulin because it consists of non-digestible carbohydrates that can stimulate the growth of bacteria that produce SCFAs [14,15] and enhance neuroprotection [16,17]. We expect the outcomes from the study will advance our knowledge on dysbiosis after CHI from acute to chronic phases, and the potential of dietary intervention for facilitating CHI recovery.

## Methods

### Experimental design

**Figure 1:**
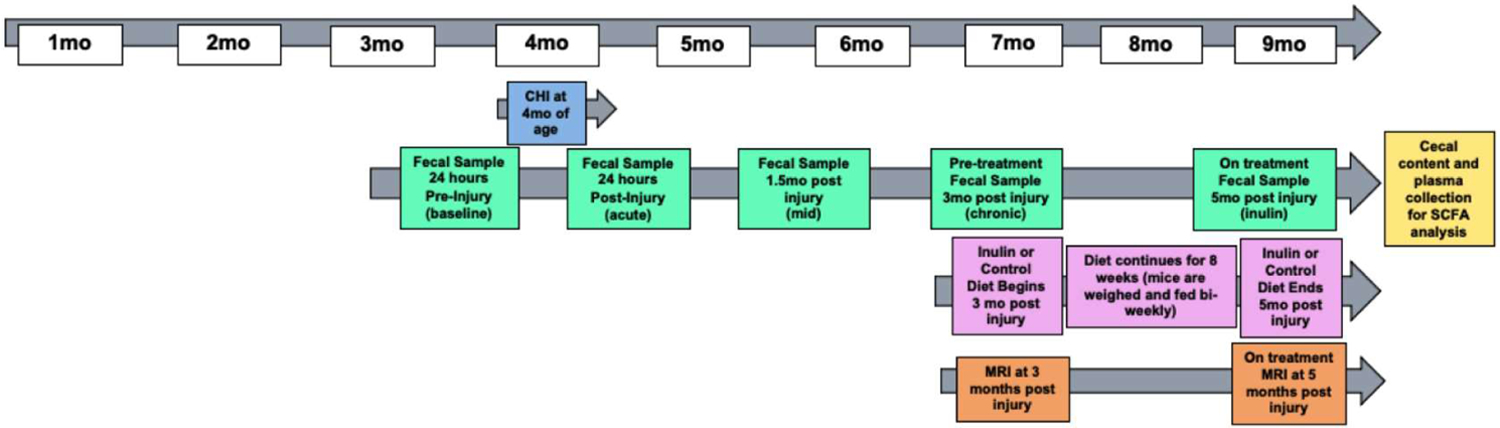
Study design. CHI was administered at 4 months of age. Diet was started 3 mpi (7 months of age) and fecal samples were taken periodically throughout the study starting 24 hours prior to injury and ending 5 mpi. CBF and FA were analyzed at 3 mpi and 5 mpi. Cecal contents and plasma were collected at time of sacrifice.

We used male C57BL/6 wild-type mice for all experiments (total N=20). Ten mice were placed in the Sham group and ten mice were placed in the closed head injury (CHI) group. Mice were singly housed throughout the study to avoid feces exchange as mice are coprophagic. Mice were administered a CHI at 4 months of age. Figure 1 shows the study design. Fecal samples were collected throughout the study at 24 hours before injury, 24 hours post injury, 1.5 mpi, 3 mpi, and 5 mpi. Before diet administration, at 3 mpi we assessed CBF and WMI (indexed by fractional anisotropy (FA)) using MRI. After this, mice were fed either a prebiotic diet containing inulin (catalog number 1817795–203) that is fermentable (Sham-Inulin, CHI-Inulin) or a control diet containing cellulose (catalog number 1817794 −203) that is nonfermentable (Sham-Control, CHI-Control) for a period of 8 weeks, or until 5 mpi (9 months of age) (n=5/group). Table 1 shows the diet composition. We fed the mice 8% inulin because 8% inulin has shown positive results on the gut microbiome and related metabolites, as well as other metrics such as brain and systemic metabolism in previous studies from our lab [14]. Inulin has a higher percentage of carbohydrates than cellulose because inulin is a fermentable fiber which means that inulin can be fermented by the gut microbiome and turned into metabolites such as SCFAs which are energy sources to the body [18]. The mice had ad libitum access to the diets, and we measured the weight of the mice and the weight of the remaining food biweekly to estimate the food intake during the feeding period. At 3 mpi we assessed CBF and FA prior to diet administration. At 5 mpi we again used multi-modal MRI metrics to assess CBF and FA. Mice were euthanized by carbon dioxide inhalation and decapitated. Cecum, blood, and brain tissues were collected thereafter for metabolomics and other biochemical analyses. All experimental procedures were performed according to NIH guidelines and approved by the Institutional Animal Care and Use Committee (IACUC) at the University of Kentucky (UK).

### Closed head injury

Briefly, as previously described [19,20], mice were anesthetized with isoflurane (4–5% induction followed by 2–3% maintenance) prior to insertion of ear bars to stabilize the head in the digital mouse stereotaxic frame (Stoelting Co., Chicago, IL). A midline sagittal scalp incision was made and a 1ml latex bulb (Fisher Scientific) was placed under the head and filled with water to displace the force during impact from the ears. An electromagnetic impactor was used to deliver a single controlled mid-line cortical impact at 5.0 ± 0.2 m/s, 100 ms dwell time, and 1.0 mm impact depth. Planned exclusion criteria included depressed skull fracture or bleeding; however, no mice were excluded from the study. Sham-injured mice underwent identical surgical procedures, but no impact was delivered. Righting-reflex was recorded as an acute neurological assessment. Within one hour after injury, animals were fully conscious and able to ambulate.

### Gut microbiome sequencing

Fecal samples were collected by putting each mouse in a clean autoclaved cage and waiting for it to defecate normally. Samples were collected during a 2-hour window from 7 am to 9 am. Samples were used to obtain genomic DNA. QIAGEN DNeasy PowerSoil Kit was used per manufacturers’ guidelines. Shotgun metagenomic sequencing was done by CosmosID. Briefly, DNA libraries were assembled using the CosmosID proprietary library prep kit and pooled by adding an equimolar ratio of each sample on a high sensitivity chip to estimate size. Libraries were then sequenced using an Illumina Miseq platform. Unassembled reads were directly analyzed by the CosmosID bioinformatics platform (CosmosID Inc., Rockville, MD), which allows for multi-kingdom microbiome analysis, profiling of antibiotic resistance and virulence genes, and quantification of relative abundance. CosmosID uses a high-performance k-mer based algorithm that disambiguates hundreds of millions of short reads of a sample into the microorganisms that the sequences represent. Matrix tables of detected taxa were generated, and heat maps were produced. These allow for visualization of diversity and abundance of each microbial taxa.

### MRI data acquisition

We used the 7T Clinscan MR scanner (Siemens, Germany) at the Magnetic Resonance Imaging & Spectroscopy Center of UK. Mice were anesthetized with 4.0% isoflurane for induction and then maintained in a 1.2% isoflurane and air mixture using a facemask. Heart rate (90–130 bpm.), respiration rate (70–90 breaths per minute), and rectal temperature (37 ± 0.5°C) were monitored. A water bath with circulating water at 45–50°C was used to maintain the body temperature. Quantitative CBF (with units of mL/g per minute) was measured using MRI-based pseudo-continuous arterial spin labeling (pCASL) techniques [21]. Paired images were acquired in an interleaved fashion with FOV=18 × 13.5 mm^2^, matrix=64 × 48, slice thickness=1 mm, 6 slices, labeling duration=200 ms, TR=4,000 ms, TE=20 ms, and 120 repetitions. pCASL image analysis was employed with in-house written codes in MATLAB (MathWorks, Natick, MA) to obtain quantitative CBF [22]. CBF maps were visualized and quantitated using Mango image viewer (UT San Antonio, TX). Diffusion tensor imaging (DTI) is used to characterize microstructural changes in the brain [23]. The images are acquired using four-segment, spin-echo, echo-planar sequence with the following parameters: field of view=22 × 14.3 mm, 160 × 160 matrix, slice thickness=1 mm, slice numbers=4, TR=1400 ms, TE=42 ms, 90 degree flip angle, *b* value=0 and 800 s/mm^2^, diffusion direction=106, diffusion gradient amplitude (G)=10 and 190 mT/m, gradient duration (Δ)=18 ms, and averages=1 [24]. Fractional Anisotropy (FA) values are analyzed using Mango image viewer for regions of interest.

### Short chain fatty acid analysis of plasma and cecal contents

Eight SCFAs from cecum and plasma were analyzed with LCMS/MS by Metabolon Inc. (Morrisville, NC): acetic acid (C2), propionic acid (C3), isobutyric acid (C4), 2-methylbutyric acid (C5), isovaleric acid (C5), valeric acid (C5), and caproic acid (C6). Both sets of samples were stable labeled with internal standards and homogenized in an organic solvent. The samples were then centrifuged followed by an aliquot of the supernatant used to derivatize to form SCFA hydrazides. This reaction mixture was subsequently diluted, and an aliquot was injected into an Agilent 1290/AB Sciex QTrap 5500 LCMS/MS system.

### Data analysis

#### Microbiome diversity and dissimilarity analyses:

Alpha diversity (within sample, Shannon index) and beta diversity (dissimilarities between samples, Bray-Curtis index) were performed in order to compare the microbial community as a whole between different sample groups (i.e. diet, injury). Data analysis and visualizations were performed by CosmosID (Rockville, MD), with Wilcoxon Rank-Sum and PERMANOVA tests. Linear Discriminant Analysis Effect Size (LEfSe) differential abundance analyses were performed between pairs of cohorts to determine taxa that are significantly enriched in each cohort.

#### Machine learning (ML) analysis:

To identify the bacterial species that play the pivotal role to differentiate dysbiosis at different timepoint in each group (i.e. Baseline vs. Acute in Sham and Acute vs. Mid in CHI), we performed ML analysis to rank the relative importance of the microbiota. We used the Support Vector Machine (SVM) algorithm for supervised classification and the SHapley Additive exPlanations (SHAP) to visualize the SVM prediction results. We applied 3-fold cross-validation and assess the algorithm’s performance with Accuracy, F1 score, and Area Under the Curve (AUC). For SVM, we used the python function “sklearn.svm.svc” to implement the SVM with hyperparameters: C=10, gamma=‘auto’, probability=True”. For SHAP, we used the function “KernelExplainer()” for the SVM model and adopted “summary_plot” functions to visualize the SVM model predictions.

### Statistical analysis

All statistical analyses were completed using GraphPad Prism 9.0 (GraphPad, San Diego, CA, USA). Two-way ANOVAs were performed for determination of differences between groups (injury type and diet effects) followed by post-hoc Welch’s t-tests. Levels of statistical significance were reached when p<0.05. For the microbiome, given the multiple comparisons inherent in analysis of microbiota, between-group relative differences are assessed using both p-value and false discovery rate analysis (q-value).

## Results

### Gut microbial beta diversity altered differently between CHI and Sham injuries

**Figure 2:**
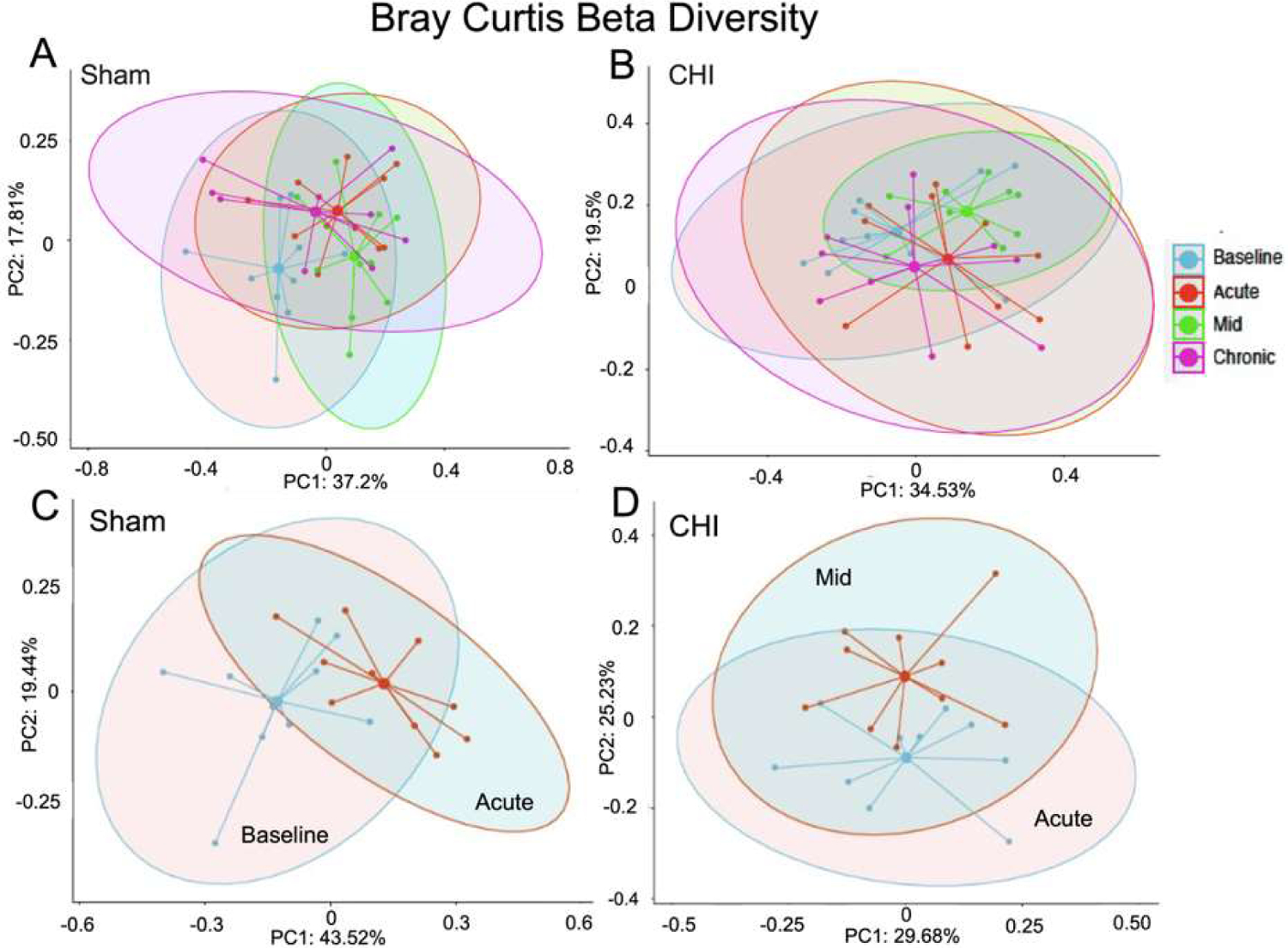
Longitudinal analysis of beta diversity. **(A)** Sham Beta diversity (*Bray-Curtis Index*) between sample longitudinal analysis. **(B)** CHI Beta diversity (*Bray-Curtis Index*) between sample longitudinal analysis. **(C)** Beta diversity showed that in the Sham group, the baseline timepoint significantly differed from the acute timepoint (Sum of Squares=0.3443181, R^2^=0.2042643, F=4.620575, p=0.003). **(D)** Beta diversity showed that in the CHI group the acute timepoint significantly differed from the mid timepoint (Sum of Squares=0.1867712, R^2^=0.127083, F=2.620517, p=0.011).

Figures 2A and 2B show the beta diversity at the four longitudinal timepoints of the Sham and CHI groups, respectively. We found significant difference within the Sham group between the Baseline and Acute timepoints (Figure 2C) (Sum of Squares=0.3443181, R^2^=0.2042643, F=4.620575, p=0.003), and within the CHI group between the Acute and Mid timepoints(Figure 2D) (Sum of Squares=0.1867712, R^2^=0.127083, F=2.620517, p=0.011).

### Bacterial species differentiate dysbiosis under Sham and CHI at different timepoints

**Figure 3:**
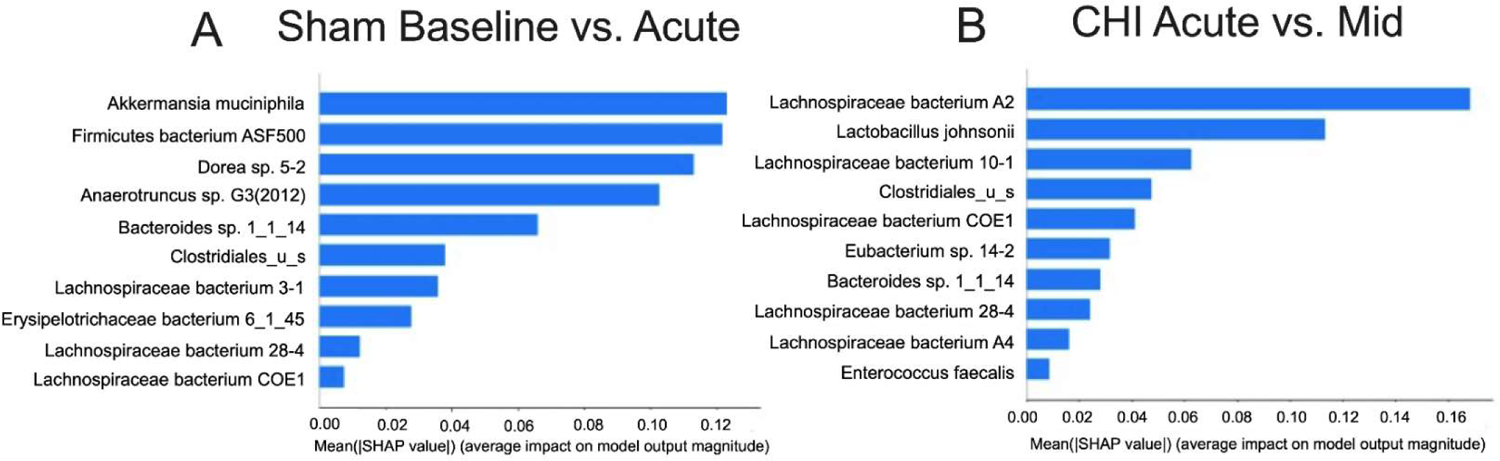
Importance ranking of microbial species. SHAP is a machine learning method that stands for SHapley Additive exPlanations **(A)** SHAP values from ML analysis indicate the top 10 most important microbiota in distinguishing the Sham Baseline vs. Acute timepoints **(B)** SHAP values indicate the top 10 features most important in distinguishing the CHI Acute vs. Mid timepoints.

Figure 3A shows the top 10 microbiota ranked by ML algorithm that can separate dysbiosis status at the Baseline from Acute timepoint in the Sham group. The ML performance was robust with 84.9% accuracy, 84.6% F1 score and 88.9% AUC. Compared to the Baseline, we found that Acute timepoint had decreases in beneficial bacteria such as *Akkermansia muciniphila* and increases in bacteria such as *Firmicutes bacterium ASF500* (Table 2). Similarly, Figure 3B shows the top 10 microbiota ranked by ML algorithm that can separate dysbiosis status at the Acute from Mid timepoint in the Sham group. The ML performance was also robust with 75.9% accuracy, 74.7% F1 score and 85.6% AUC. Compared to the Acute phase, we found that the Mid timepoint had decreases in beneficial bacteria such as *Lactobacillus johnsonii*, and increases in species from the *Lachnospiraceae* family, which is known to be associated with various disease states, such as *Lachnospiraceae bacterium A*2, *28–4* and *A4*. Table 2 shows the relative abundance of the species in the importance table for each timepoint, with lower values shaded in gray.

### CHI mice show decreased CBF and changes in FA compared to Sham mice

**Figure 4:**
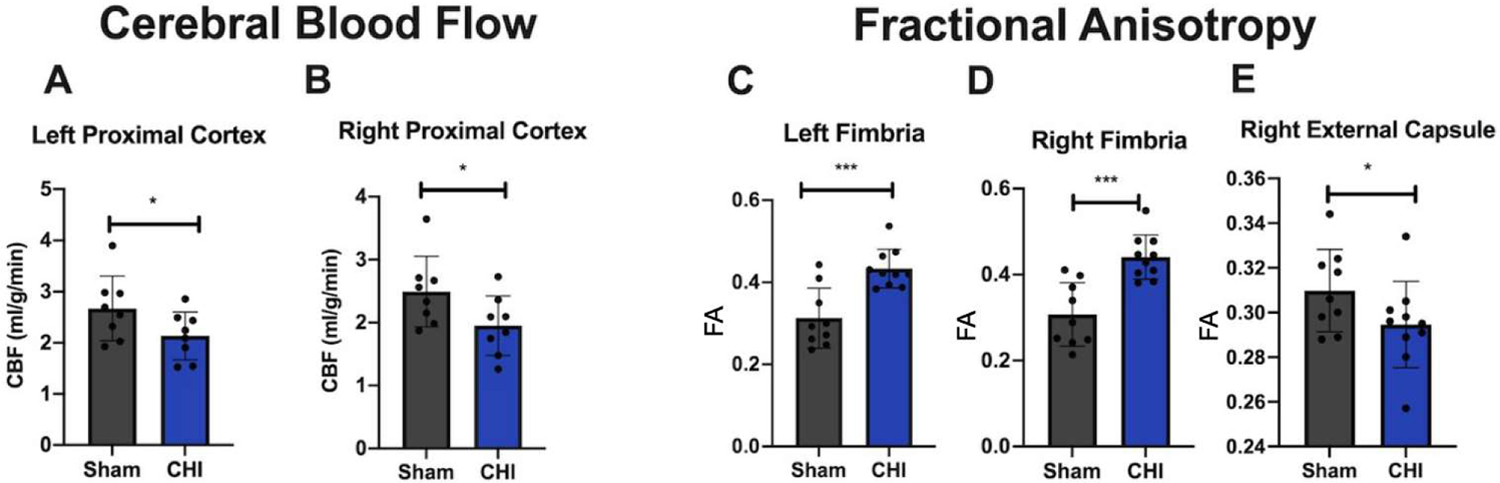
Cerebral blood flow and fractional anisotropy analysis. **(A-B)** CHI decreases CBF in the **(A)** left proximal cortex, and **(B)** right proximal cortex compared to Sham **(C-E)** CHI increases FA in the **(C)** left fimbria and **(D)** right fimbria and decreases FA in the **(E)** right external capsule compared to Sham. Data are presented as mean ± SD. *p<0.05, ***p<0.001.

CBF and FA were measured 3 mpi. CBF was shown to be lower in the CHI group compared to the Sham group in the left (Figure 4A) and right proximal cortex (Figure 4B). There are higher levels of FA in the left (Figure 4C) and right fimbria (Figure 4D) compared to the Sham group and lower levels of FA in the right external capsule (Figure 4E) compared to the Sham group. Changes in FA fit with the literature which indicates that decreased FA is expected after injury, but FA can also be increased after injury potentially due to compensatory mechanisms or edema [25].

## Post-Diet Results

### Inulin altered gut microbial diversity in both Sham and CHI mice

**Figure 5:**
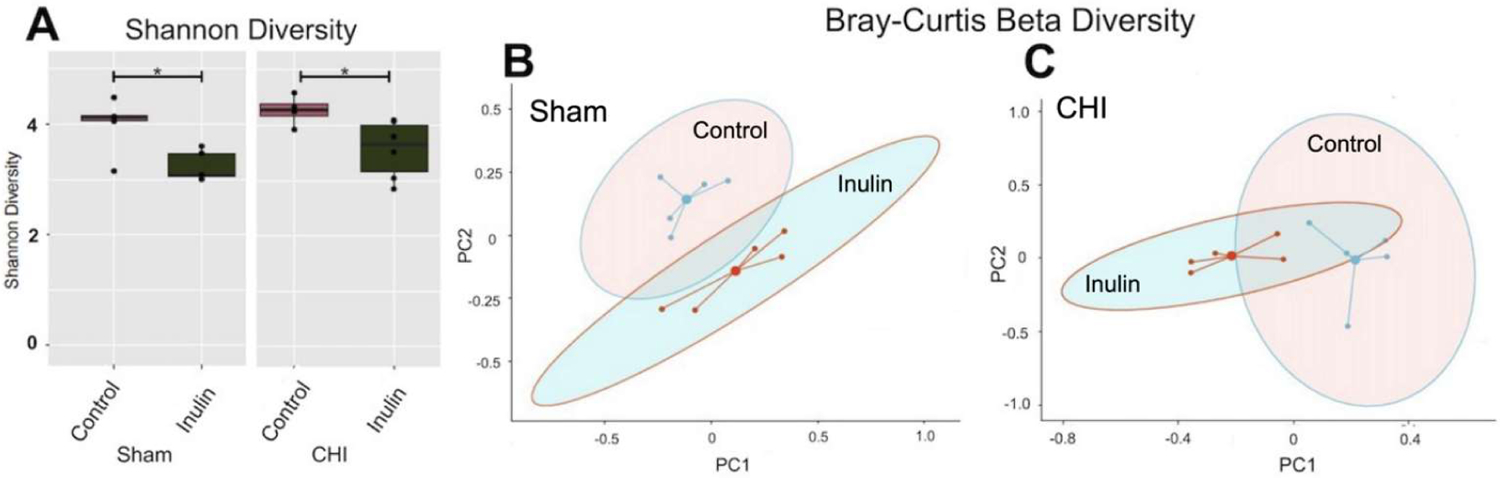
Gut microbiome diversity. **(A)** Alpha diversity (*Shannon index*) within sample analysis shows that inulin decreased alpha diversity in Sham and CHI, (*p ≤ 0.05). **(B)** Beta diversity (*Bray-Curtis Index*) between sample analysis showed that Sham inulin fed mice had a significantly different diversity than Sham control fed mice (Sum of Squares= 0.3417861, R^2^=0.2355275, F=2.464733, p=0.038). **(C)** Beta diversity (*Bray-Curtis Index*) between sample analysis showed that CHI inulin fed mice had a significantly different diversity than CHI control fed mice, (Sum of Squares=0.4846131, R^2^=0.3702754, F=4.703967, p=0.012).

After two months of inulin feeding at the chronic phase, we found that alpha diversity was reduced in both the Sham and CHI groups (p<0.05) (Figure 5A). Similarly, inulin significantly altered beta diversity in the Sham (Figure 5B) (Sum of Squares=0.3417861, R^2^=0.2355275, F=2.464733, p=0.038) and CHI groups (Figure 5C) (Sum of Squares=0.4846131, R^2^=0.3702754, F=4.703967, p=0.012) using the Bray-Curtis index.

### Inulin altered gut microbiome at the phylum level predominately in the CHI mice

**Figure 6:**
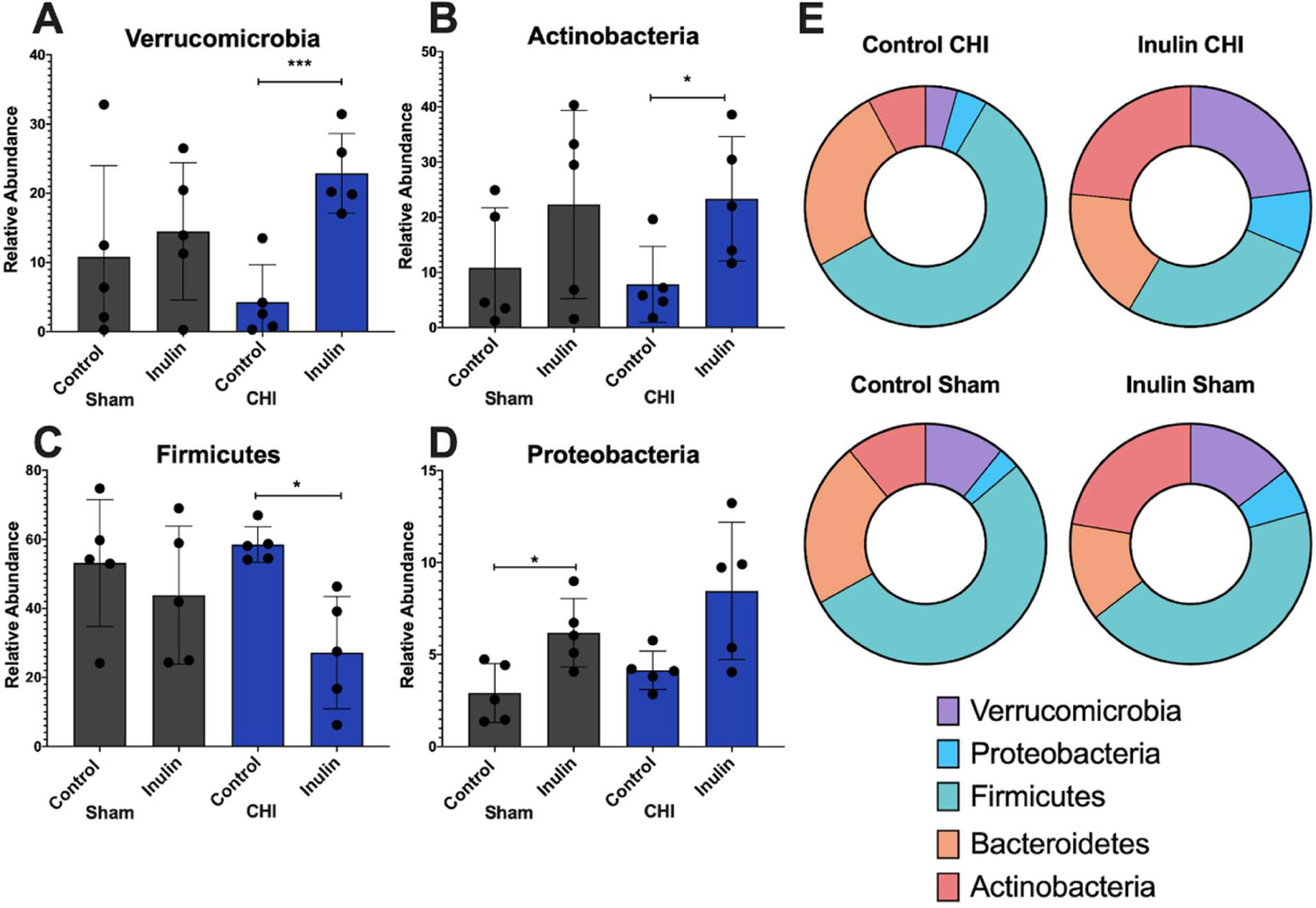
Phyla level analysis of gut microbiome. **(A-D)** Quantitative analysis of phyla. Inulin showed increases in the CHI group for the phyla **(A)** *Verrucomicrobia* and **(B)** *Actinobacteria* and decreases in the CHI group for the phyla **(C)** *Firmicutes*. Inulin showed an increase in the Sham group for the phyla **(D)** *Proteobacteria*. **(E)** Qualitative analysis of phyla to visualize patterns of changes. Data are presented as mean ± SD. *p<0.05, ***p<0.001.

At the phylum level, we found that in the CHI group, inulin increased *Verrucromicrobia* (p=0.0008) (Figure 6A), and *Actinobacteria* (p=0.0359) (Figure 6B) but decreased *Firmicutes* (p=0.0102) (Figure 6C) compared to the CHI control mice. Inulin significantly increased *Proteobacteria* in the Sham group (p=0.0178) but not the CHI group (p=0.0590) (Figure 6D). No significant differences of *Bacteroidetes* were found between the control and inulin diet in both CHI and Sham groups (Sham control vs. inulin p=0.1978; CHI control vs. inulin p=0.2691). The distribution of the relative abundance of the five phyla is shown in Figure 6E.

### Inulin increased SCFA-producing bacteria and reduced pathogenic bacteria in CHI mice

**Figure 7:**
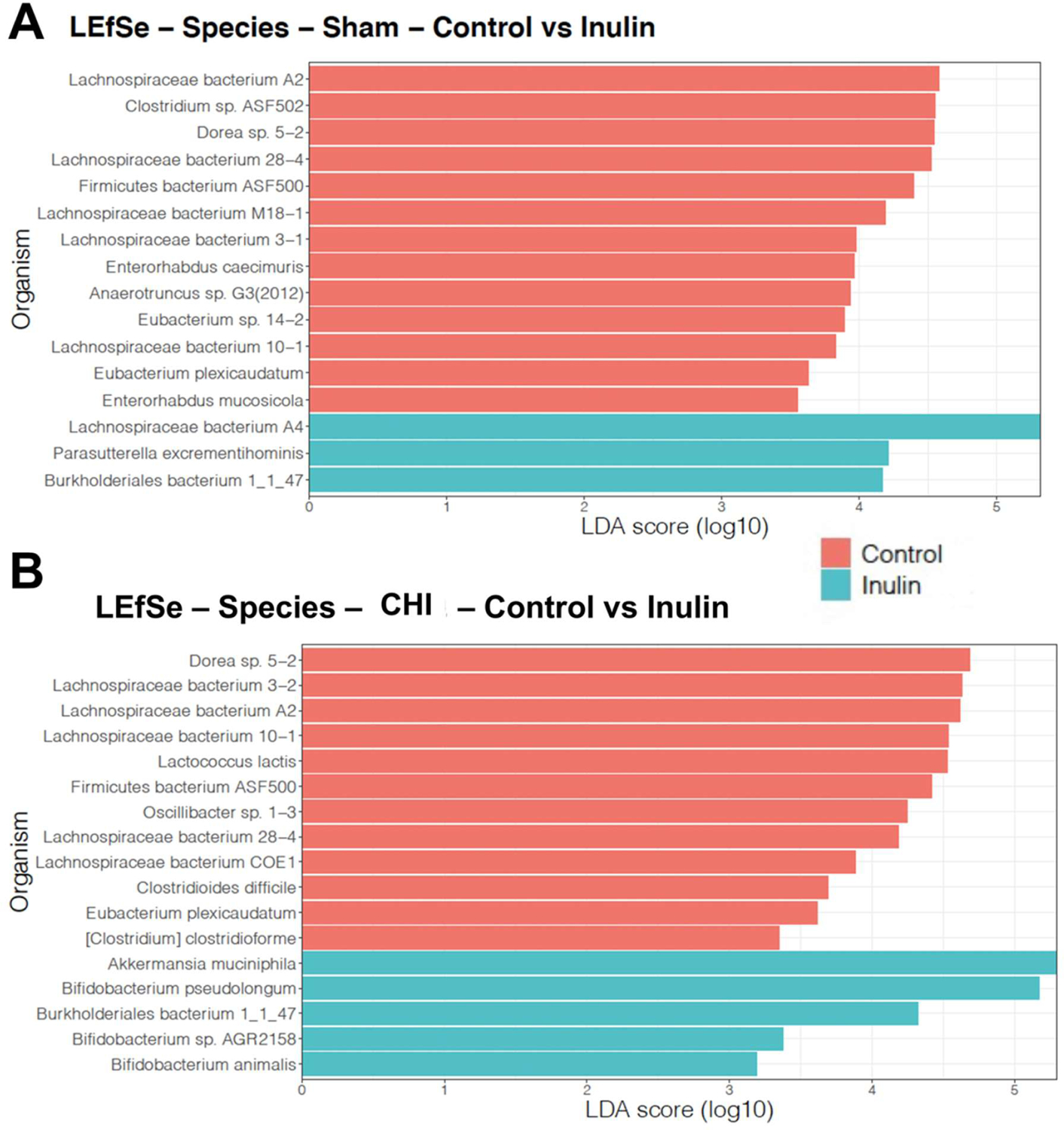
Species-level analysis of the gut microbiome. **(A)** Analysis at the species level of Sham control versus Sham inulin mice. **(B)** Analysis at the species level of CHI control versus CHI inulin fed mice. Analysis was conducted using Linear discriminant analysis Effect size (LEfSe). Control-fed CHI or Sham mice are shown in peach and inulin-fed CHI or Sham mice are shown in teal. Bars indicate that a specific species was found to be significantly increased with the diet specified. All shown are statistically significant (p ≤ 0.05) (LDA ≥ 2.0).

Using LEfSe analysis, we determined taxonomic similarities and differences between Sham (Figure 7A) and CHI mice (Figure 7B) at the species level. The control diet selected for species in the CHI and Sham groups including the *Lachnospiraceae* family and *Clostridium* and *Clostridioides* genera, as well as other species such as *Dorea sp. 5–2* and *Oscillibacter sp. 1-*3. However, the species selected for inulin differ between the Sham and CHI groups. In the CHI group, but not the Sham group, inulin promoted several bacteria that have a positive effect on the gut and produce beneficial metabolites such as SCFAs including *Bifidobacterium* species (*animalis, AGR1258, pseudolongum)* and *Akkermansia muciniphila*.

**Figure 8:**
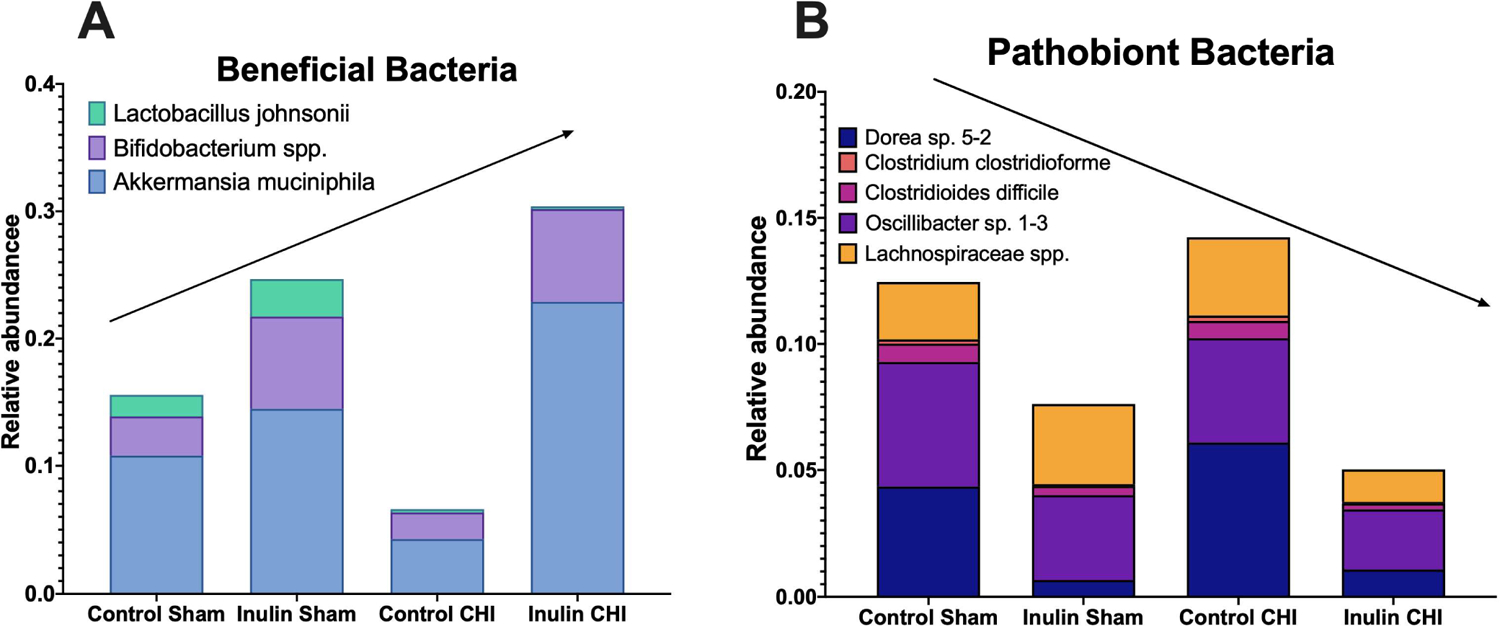
Inulin increases beneficial bacteria and decreases pathobiont bacteria. **(A)** Inulin increases beneficial bacteria *Bifidobacterium spp, Lactobacillus johnsonii and Akkermansia muciniphila*. **(B)** Inulin decreases pathobiont bacteria *Dorea sp. 5–2, Clostridium clostridioforme, Clostridioides difficile, Oscillibacter sp. 1–3*, and *Lachnospiraceae spp*. Significant bacteria were chosen from the LEfSe analysis (p ≤ 0.05).

The major changes at the species levels are summarized in Figure 8. Based on the LEfSe analysis, we found that inulin increased putatively beneficial bacteria (Figure 8A) and decreased pathobiont bacteria (Figure 8B). Compared to the control diet, the inulin diet decreased *Dorea sp. 5–2* and *Oscillibacter sp. 1–3*, which have both been positively correlated with intestinal permeability [26,27]; *Clostridium clostridioforme*, which is associated with bacteriemia [28]; and *Clostridioides difficile*, a pathobiont [29]. Inulin also decreased *Lachnospiraceae spp*. (including *L. bacterium 3–2, L. bacterium A2, L. bacterium 10–1, L. bacterium 28–4, and L. bacterium COE1*) which have been implicated in colitis [30] and metabolic disorders [31]. Inulin increased *Bifidobacterium pseudolongum*, a SCFA producer*, Bifidobacterium animalis* and *Bifidobacterium sp. AGR2158*, which fits with the literature indicating inulin as a bifidogenic fiber [32]. It also increased *Akkermansia muciniphila*, which is also a SCFA producer [33]. *Lactobacillus johnsonii* does show a decrease in the CHI group, but this is likely due to out-competition from *Bifidobacterium* spp*. and A. muciniphila*.

### Inulin increased cecal and serum SCFA levels in the CHI and Sham mice

**Figure 9:**
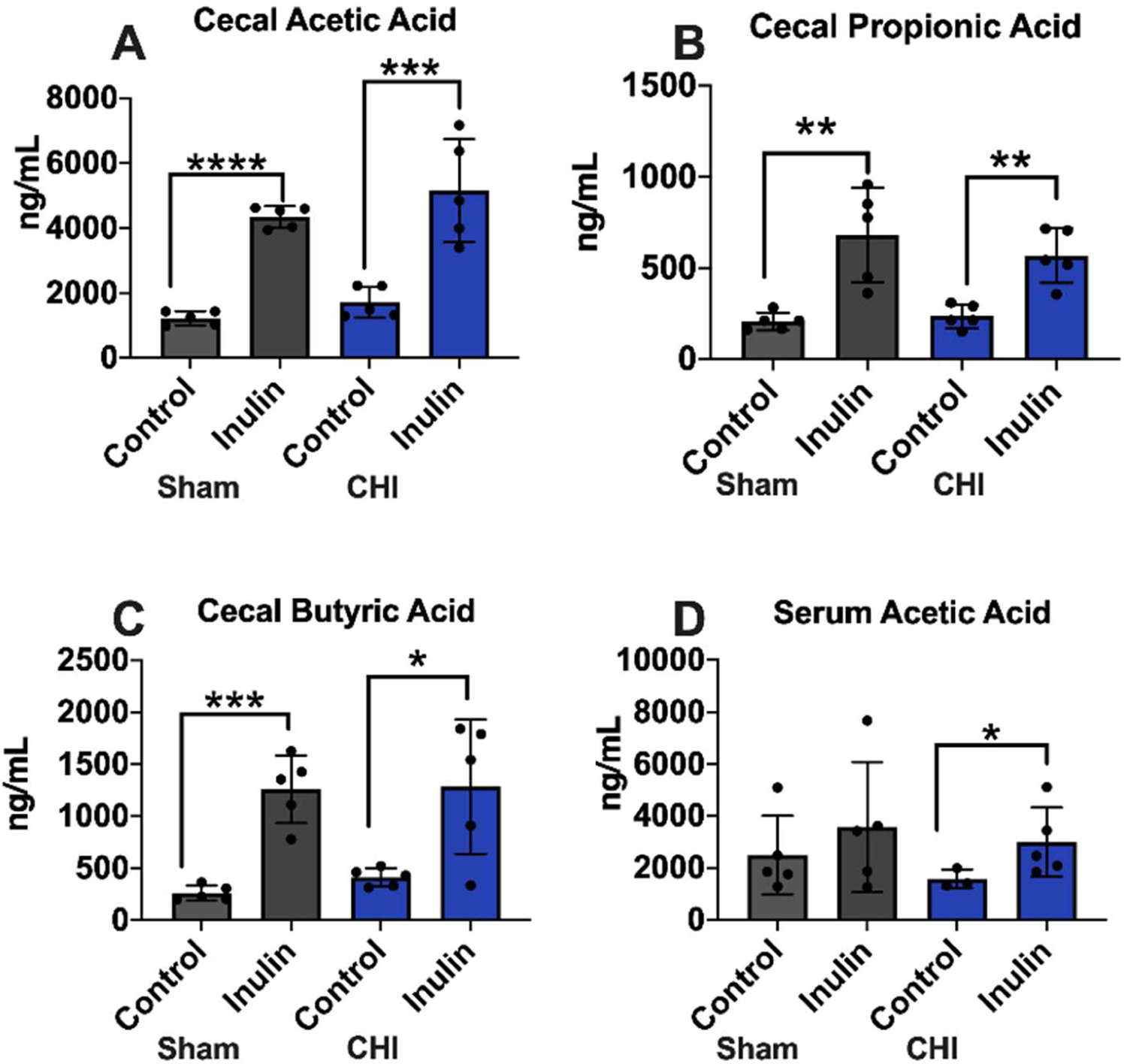
Short Chain Fatty Acids (SCFAs) were increased with inulin. Cecal levels SCFAs were increased in Sham and CHI groups with inulin including **(A)** acetic acid, **(B)** propionic acid, and **(C)** butyric acid. Serum levels of **(D)** acetic acid were also increased with inulin. Data are presented as mean ± SD. *p<0.05, **p<0.01, ***p<0.001, ****p<0.0001.

In the cecum SCFA analysis, most notably, the inulin-fed groups (Sham and CHI) showed significant increases in acetic acid (Sham p<0.0001; CHI p=0.0032) (Figure 9A), propionic acid (Sham p=0.0070; CHI p=0.0024) (Figure 9B), and butyric acid (Sham p=0.0009; CHI p=0.0193) (Figure 9C) compared to the control fed groups. In the serum, only acetic acid was found significantly different between the CHI-control and CHI-inulin mice (p=0.0385) (Figure 9D).

### Inulin restored CBF after CHI

**Figure 10:**
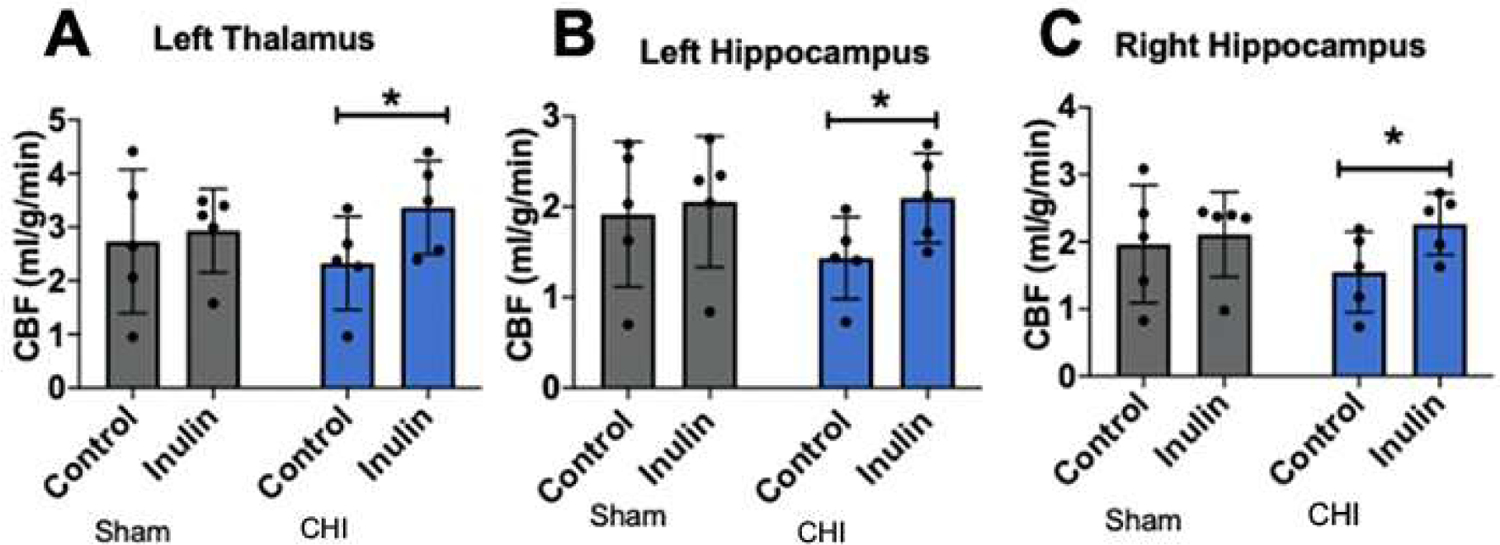
Inulin increases cerebral blood flow after CHI. **(A-C)** Inulin increased CBF in the **(A)** left thalamus, **(B)** left hippocampus, **(C)** right hippocampus compared to controls. Data are presented as mean ± SD. *p<0.05.

We did not observe injury type- or diet-dependent CBF changes at 5 mpi (injury p=0.4471, F(1,16)=0.6076; diet p=0.1783, F(1,16)=1.982). However, with post-hoc analysis, we did find higher CBF in inulin-fed vs. control-fed CHI mice in the left thalamus (p=0.0482) (Figure 10A), left hippocampus (p=0.0292) (Figure 10B) and right hippocampus (p=0.0343) (Figure 10C).

## Discussion

**Figure 11:**
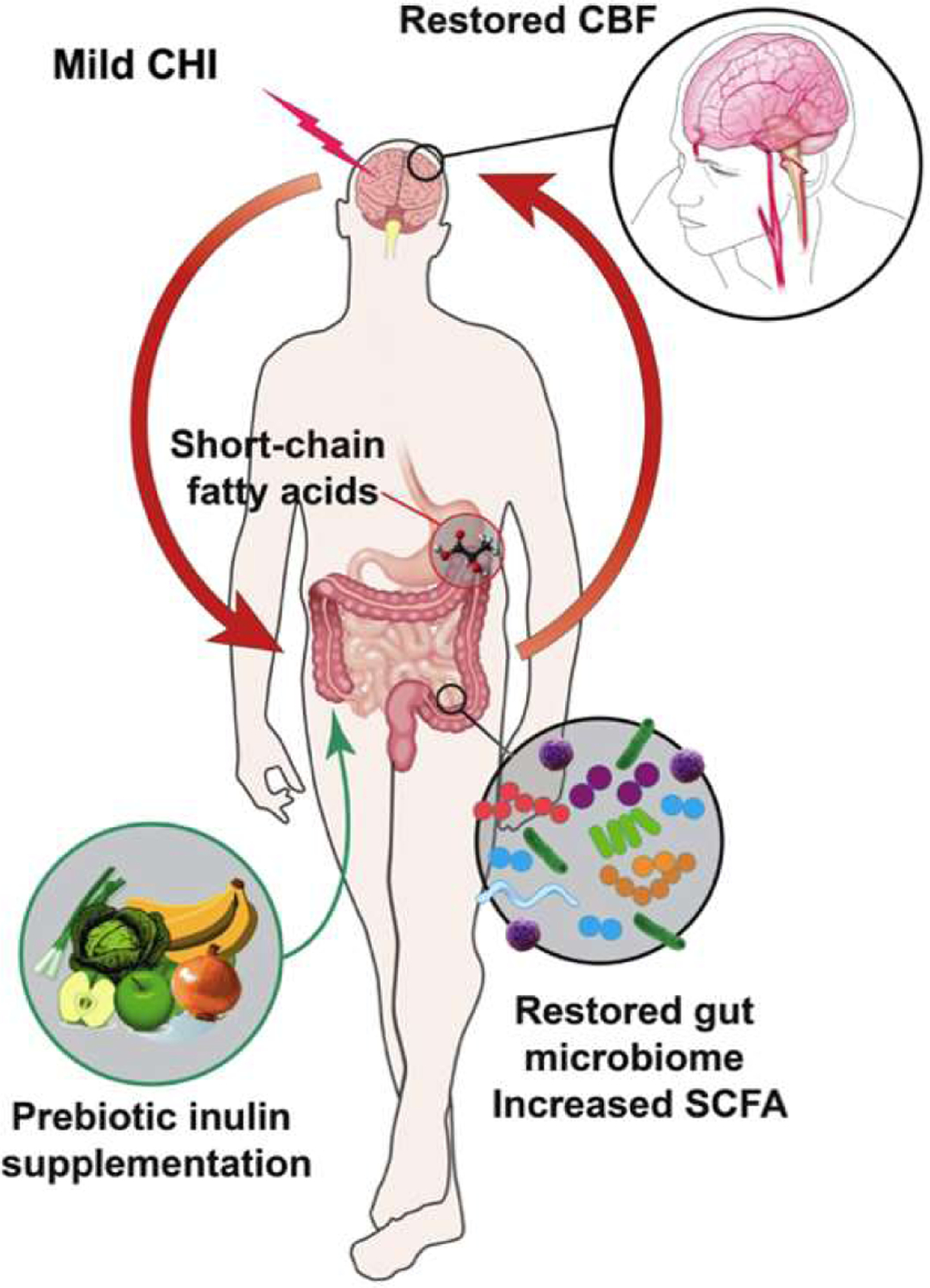
Summary of findings. CHI induced gut dysbiosis and disrupted cerebral blood flow and brain structural metrics. With prebiotic inulin supplementation at 3 mpi, results show an increase of putatively beneficial bacteria and a decrease of pathobiont bacteria, an increase in SCFAs and restored CBF.

To our knowledge, our study is the first to characterize the gut microbiome in the chronic phase (past one week post CHI) [34,35]. We further showed that CBF and WMI of CHI mice were impacted in the acute phase but normalized to a level similar to that of the Sham mice in the chronic phase. We summarized our findings in Figure 11. It shows that despite the CHI-induced dysbiosis, prebiotic inulin was able to increase the level of beneficial bacteria, reduce pathobiont bacteria, and elevate SCFAs in the cecal contents and plasma. Inulin also restored CBF in both hippocampi and the left thalamus. Inulin may mitigate long-term effects of mTBI due to positive effects on the gut microbiome.

An interesting finding from the study is that the Sham mice also showed dysbiosis at different timepoint compared with the CHI mice. This is unexpected based upon data from previous studies [36]. Sham mice showed dysbiosis at the Baseline-Acute phase, while CHI mice showed dysbiosis at the Acute-Mid phase. The top three species that classify Baseline vs. Acute timepoints in the Sham group were decreased *Akkermansia muciniphila*, increased *Firmicutes ASF500*, and decreased *Dorea sp. 5–2*. Decreased *Akkermansia muciniphila* has been observed in patients with ulcerative colitis and other metabolic disorders, indicating its potential anti-inflammatory properties [37]. Increases are seen in *Firmicutes ASF500* levels with excess manganese exposure, which is neurotoxic and can lead to Alzheimer’s and Parkinson’s Disease [38]. Decreased levels of *Dorea sp. 5–2* are beneficial as this species is associated with intestinal permeability [26], indicating the possible transient nature of dysbiosis in the Sham group. Although there was some dysbiosis (increased *ASF500*, decreased *Akkermansia muciniphila*), there was also some recovery (decreased *D. sp. 5–2*). As for the CHI group, between the Acute to Mid timepoint, the most important feature was the increased *Lachnospiraceae bacterium A2*. The *Lachnospiraceae* family is associated with colitis [30] and metabolic disorders, such as Type 2 Diabetes, metabolic syndrome, and glucose metabolism disorders [31]. Another top feature was decreased *Lactobacillus johnsonii*, which has probiotic properties such as immunomodulation and pathogen inhibition. Therefore, its decrease could be indicative of a dysbiotic state [39].

With diet, we found significant decreases in alpha diversity in the inulin-fed mice compared to the control-fed mice, in both Sham and CHI groups. It is likely due to inulin serving as a dominant energy source and selecting for a limited number of putatively beneficial bacteria, and an overall reduction in alpha diversity. This is similar to what we observed in a previous study [14]. At species level, we observed that inulin decreased putatively harmful bacteria compared to the mice fed the control diet. *Dorea sp. 5–2* and *Oscillibacter sp. 1–3* have both been shown to be positively correlated with intestinal permeability [26,27] and are reduced with inulin supplementation. Inulin also showed decreased *Lachnospiraceae spp*. compared to the control diet, which is associated with various disease states [31]. Along with this, inulin decreased levels of *Clostridioides difficile*, a common opportunistic nosocomial infection that blooms when the gut is in a dysbiotic state, such as after antibiotic administration [29]. We speculate that the dysbiotic state of the gut microbiome following CHI allowed for *C. difficile* to bloom, but inulin, which increased bacteria with anti-pathogenic qualities, such as *Bifidobacterium pseudolongum*, then decreased its levels [14].

The putatively beneficial bacteria such as *Akkermansia muciniphila* and *Bifidobacterium pseudolongum* are known to be anti-inflammatory in nature, as both are producers of SCFAs -- *B. pseudolongum* produces butyrate while *Akkermansia muciniphila* produces acetate -- both of which were increased in CHI mice fed inulin [12]. Butyrate and other SCFAs may be able to inhibit *C. difficile* overgrowth by causing the bacteria to lose viability [40,41]. As we see increased SCFAs and decreased *C. difficle* in inulin-fed mice, this could be a possible protective mechanism of inulin supplementation from increased *C. difficile* levels after injury. It is also known that a low abundance of *Akkermansia muciniphila* favors the inflammatory process [33], and we saw a much lower abundance of this bacteria in the control-fed CHI group compared to the inulin-fed CHI group. The increased levels of *Akkermansia muciniphila* in the inulin group is also consistent with the literature in that this species thrives on fiber [33].

We found decreased white matter integrity (indexed by FA) in the chronic phase of CHI. FA was lower in the external capsule, which is the route for cholinergic fibers from the forebrain to the cerebral cortex [42]. Higher FA values were seen in the fimbria, which is the major output tract of the hippocampus, where damage can cause difficulty in long term recall [43]. This is possibly due to a neuroplastic response to injury because compensatory mechanisms are most active during the acute and subacute periods of injury [25]. However, these higher values could also be due to axonal swelling and edema in the brain [25]. Interestingly, at 5 mpi, we did not see the FA difference between the Sham control vs. CHI control group that we saw at 3 mpi. (see Figure 4), which may indicate a normalization of white matter structure. This fits with the literature that FA typically recovers between the acute and subacute periods of injury recovery [44].

Similar to the pattern found in FA, we also observed that CBF decreased at 3 mpi, but normalized at 5 mpi, in the CHI mice compared with the Sham mice. The decreased CBF was found in the proximal cortex on the right and left side, which is the area adjacent to the injury.

Nonetheless, inulin supplementation in CHI mice restored CBF to Sham levels in the left side of the thalamus and the left and right sides of the hippocampus. The thalamus plays an important role in relaying sensory and motor signals and regulates consciousness and alertness. This is consistent with a recent study showing that supplementation with a diet rich in fruit and vegetables attenuated neuromotor and sensorimotor functions following TBI [45]. It is also possible that inulin influenced CBF through increased SCFAs. As shown, there were increases in SCFAs with inulin supplementation, butyrate and propionate in the cecum and acetate in the cecum and blood. SCFAs are known to increase tight junction proteins in the gut and brain which leads to less permeable barriers [12]. A less permeable blood-brain barrier due to increased tight junction protein expression is protective of blood flow [46]. The gut microbiome is directly related to this, as *Bifidobacterium pseudolongum* is a butyrate producer and *Akkermansia muciniphila* is an acetate producer. Both were increased with inulin supplementation [32]. Previous studies from our lab are indicative of this as well [46]. A ketogenic diet given to young healthy mice increased CBF compared to the control diet and showed similarities in the gut microbiome to our study with decreased *Clostridium* spp. and *Dorea* spp. and increased *Akkermansia muciniphila* [46] *. Dorea* spp. and *Clostridium* spp. are both known to increase intestinal permeability [26,47] whereas *Akkermansia muciniphila* is known to protect the gut barrier and produces SCFAs which are also protective of the blood brain barrier, and ultimately CBF. Together, changes in abundance of these three species may facilitate protection of neurovascular functions and may be indicative of why we saw a restoration of CBF after CHI.

This study explores changes in the gut microbiome past the acute phase of injury and sheds new light onto the chronic phase of injury recovery. Even when administered three months post injury, inulin’s effects still had a positive impact on recovery through attenuating harmful effects caused by CHI in the gut microbiome, systemic metabolism, and brain vascular and structural function. This is in line with previous findings that protecting brain metabolism is important to mitigate CNS injury and age-related neurodegenerative disorders [6,48–55]. In the future, we could also use other MRI neuroimaging methods to determining brain metabolic and vascular functions that may be influenced by inulin, including levels of essential brain metabolites [56], cerebral metabolic rate of oxygen [57,58], and cerebral blood volume [59].

A limitation of the study is that we only used male mice because men are more likely to avoid treatment or not report the head injury at all, contributing to the high number of undiagnosed injuries [60]. In the future studies, we will include female mice and further identify the potential sex-differences in response to the diet. We will also include analyses to understand how inulin is exerting its effects on a molecular level. Additionally, the Sham injury causing transient changes in the gut microbiome indicates the importance of including a naïve cohort in injury studies that aim to better understand changes in the gut microbiome. It is the standard in mTBI research to use Sham mice as a control group, however this study proves that naïve mice are needed to properly assess effects of injury.

In summary, we found that CHI induces dysbiosis and alterations in WMI and CBF through the chronic phase of injury. Additionally, we found that inulin supplementation exerted a beneficial effect on the gut microbiome which in turn led to beneficial influence systemically, most pertinently on SCFAs levels in the cecal contents and plasma, and CBF in hippocampus and thalamus in mice with chronic CHI. As there are currently no effective treatments for mTBI, the study may have profound implication for developing therapeutics or preventive interventions in the future.

## Figures and Tables

**Table 1:** Diet Composition.

Diet	Prebiotic Diet	Control Diet
Protein %	18.2	18.2
Carbohydrate %	59.1 (w/o inulin) 8.9 from inulin	60.2 (w/o inulin)
Fat %	7.1	7.1
Fiber %	8.0 (Inulin)	8.0 (Cellulose)
Energy (kcal/g)	4.08	3.78

Comparison of diet composition of the Prebiotic (inulin) diet and Control (cellulose) diet.

**Table 2:** Relative abundance of ranked microbial species.

Sham	Relative Abundance	
Species	Baseline Sham	Acute Sham
Akkermansia muciniphila	0.051286	0.009855
Firmicutes bacterium ASF500	0.017068	0.025392
Dorea sp. 5–2	0.046402	0.033166
Anaerotruncus sp. G3(2012)	0.012344	0.023688
Bacteroides sp. 1_1_14	0.04354	0.018108
Clostridales_u_s	0.064976	0.125705
Lachnospiraceae bacterium 3–1	0.019942	0.020895
Erysipelotrichaceae bacterium 6_1_45	0.001124	0.002352
Lachnospiraceae bacterium 28–4	0.009306	0.011209
Lachnospiraceae bacterium COE1	0.013027	0.015841
CHI	Relative Abundance	
Species	Acute CHI	Mid CHI
Lachnospiraceae bacterium A2	0.047835	0.115548
Lactobacillus johnsonii	0.062292	0.035032
Lachnospiraceae bacterium 10–1	0.011855	0.009426
Clostridiales_u_s	0.104587	0.07117
Lachnospiraceae bacterium COE1	0.012514	0.009342
Eubacterium sp. 14–2	0.006622	0.004595
Bacteroides sp. 1_1_14	0.022325	0.063441
Lachnospiraceae bacterium 28–4	0.009776	0.007619
Lachnospiraceae bacterium A4	0.166382	0.090773
Enterococcus faecalis	0.023502	0.015995

Comparison of relative abundance from ranked microbial species in Figure 4. Lower values are shaded in gray.

## References

[1] Bodnar CN, Roberts KN, Higgins EK, Bachstetter AD. A Systematic Review of Closed Head Injury Models of Mild Traumatic Brain Injury in Mice and Rats. J Neurotrauma. 2019;36(11):1683–706. 30661454 10.1089/neu.2018.6127 PMC6555186

[2] Carroll LJ, Cassidy JD, Peloso PM, Borg J, von Holst H, Holm L, Prognosis for mild traumatic brain injury: results of the WHO Collaborating Centre Task Force on Mild Traumatic Brain Injury. J Rehabil Med. 2004(43 Suppl):84–105. 15083873 10.1080/16501960410023859

[3] Center for Disease Control and Prevention NCfIPaC. Report to Congress on Mild Traumatic Brain Injury in the United States: Steps to Prevent a Serious Public Health Problem.. CDC: Atlanta, GA. 2003.

[4] Furlan JC, Radan MM, Tator CH. A Scoping Review of Registered Clinical Studies on Mild Traumatic Brain Injury and Concussion (2000 to 2019). Neurosurgery. 2020;87(5):891–9. 32415848 10.1093/neuros/nyaa151

[5] Bonne O, Gilboa A, Louzoun Y, Kempf-Sherf O, Katz M, Fishman Y, Cerebral blood flow in chronic symptomatic mild traumatic brain injury. Psychiatry Res. 2003;124(3):141–52. 14623066 10.1016/s0925-4927(03)00109-4

[6] Lyons DN, Vekaria H, Macheda T, Bakshi V, Powell DK, Gold BT, A Mild Traumatic Brain Injury in Mice Produces Lasting Deficits in Brain Metabolism. J Neurotrauma. 2018;35(20):2435–47. 29808778 10.1089/neu.2018.5663 PMC6196750

[7] Messe A, Caplain S, Paradot G, Garrigue D, Mineo JF, Soto Ares G, Diffusion tensor imaging and white matter lesions at the subacute stage in mild traumatic brain injury with persistent neurobehavioral impairment. Hum Brain Mapp. 2011;32(6):999–1011. 20669166 10.1002/hbm.21092 PMC6870077

[8] Urban RJ, Pyles RB, Stewart CJ, Ajami N, Randolph KM, Durham WJ, Altered Fecal Microbiome Years after Traumatic Brain Injury. J Neurotrauma. 2020;37(8):1037–51. 31868094 10.1089/neu.2019.6688

[9] Wang S, Zhu K, Hou X, Hou L. The association of traumatic brain injury, gut microbiota and the corresponding metabolites in mice. Brain Res. 2021;1762:147450. 33773978 10.1016/j.brainres.2021.147450

[10] Bai L, Li T, Zhang M, Wang S, Gan S, Jia X, Association of gut microbiota with cerebral cortex and cerebrovascular abnormality in human mild traumatic brain injury. bioRxiv. 2020.

[11] Li H, Sun J, Du J, Wang F, Fang R, Yu C, Clostridium butyricum exerts a neuroprotective effect in a mouse model of traumatic brain injury via the gut-brain axis. Neurogastroenterol Motil. 2018;30(5):e13260. 29193450 10.1111/nmo.13260

[12] Dalile B, Van Oudenhove L, Vervliet B, Verbeke K. The role of short-chain fatty acids in microbiota-gut-brain communication. Nat Rev Gastroenterol Hepatol. 2019;16(8):461–78. 31123355 10.1038/s41575-019-0157-3

[13] Sundman MH, Chen NK, Subbian V, Chou YH. The bidirectional gut-brain-microbiota axis as a potential nexus between traumatic brain injury, inflammation, and disease. Brain Behav Immun. 2017;66:31–44. 28526435 10.1016/j.bbi.2017.05.009

[14] Hoffman JD, Yanckello LM, Chlipala G, Hammond TC, McCulloch SD, Parikh I, Dietary inulin alters the gut microbiome, enhances systemic metabolism and reduces neuroinflammation in an APOE4 mouse model. PLoS One. 2019;14(8):e0221828. 31461505 10.1371/journal.pone.0221828 PMC6713395

[15] Yanckello LM, Hoffman JD, Chang YH, Lin P, Nehra G, Chlipala G, Apolipoprotein E genotype-dependent nutrigenetic effects to prebiotic inulin for modulating systemic metabolism and neuroprotection in mice via gut-brain axis. Nutr Neurosci. 2021:1–11. 10.1080/1028415X.2021.1889452 PMC8799401 33666538

[16] Huuskonen J, Suuronen T, Nuutinen T, Kyrylenko S, Salminen A. Regulation of microglial inflammatory response by sodium butyrate and short-chain fatty acids. Br J Pharmacol. 2004;141(5):874–80. 14744800 10.1038/sj.bjp.0705682 PMC1574260

[17] Bourassa MW, Alim I, Bultman SJ, Ratan RR. Butyrate, neuroepigenetics and the gut microbiome: Can a high fiber diet improve brain health? Neurosci Lett. 2016;625:56–63. 26868600 10.1016/j.neulet.2016.02.009 PMC4903954

[18] Dahl WJ, Stewart ML. Position of the Academy of Nutrition and Dietetics: Health Implications of Dietary Fiber. J Acad Nutr Diet. 2015;115(11):1861–70. 26514720 10.1016/j.jand.2015.09.003

[19] Webster SJ, Van Eldik LJ, Watterson DM, Bachstetter AD. Closed head injury in an age-related Alzheimer mouse model leads to an altered neuroinflammatory response and persistent cognitive impairment. J Neurosci. 2015;35(16):6554–69. 25904805 10.1523/JNEUROSCI.0291-15.2015 PMC4405562

[20] Bachstetter AD, Webster SJ, Goulding DS, Morton JE, Watterson DM, Van Eldik LJ. Attenuation of traumatic brain injury-induced cognitive impairment in mice by targeting increased cytokine levels with a small molecule experimental therapeutic. J Neuroinflammation. 2015;12:69. 25886256 10.1186/s12974-015-0289-5 PMC4396836

[21] Muir ER, Shen Q, Duong TQ. Cerebral blood flow MRI in mice using the cardiac-spin-labeling technique. Magn Reson Med. 2008;60(3):744–8. 18727091 10.1002/mrm.21721 PMC2581653

[22] Lin AL, Zhang W, Gao X, Watts L. Caloric restriction increases ketone bodies metabolism and preserves blood flow in aging brain. Neurobiol Aging. 2015;36(7):2296–303. 25896951 10.1016/j.neurobiolaging.2015.03.012 PMC4457572

[23] Alexander AL, Lee JE, Lazar M, Field AS. Diffusion tensor imaging of the brain. Neurotherapeutics. 2007;4(3):316–29. 17599699 10.1016/j.nurt.2007.05.011 PMC2041910

[24] Guo J, Bakshi V, Lin AL. Early Shifts of Brain Metabolism by Caloric Restriction Preserve White Matter Integrity and Long-Term Memory in Aging Mice. Front Aging Neurosci. 2015;7:213. 26617514 10.3389/fnagi.2015.00213 PMC4643125

[25] Lipton ML, Kim N, Park YK, Hulkower MB, Gardin TM, Shifteh K, Robust detection of traumatic axonal injury in individual mild traumatic brain injury patients: intersubject variation, change over time and bidirectional changes in anisotropy. Brain Imaging Behav. 2012;6(2):329–42. 22684769 10.1007/s11682-012-9175-2

[26] Leclercq S, Matamoros S, Cani PD, Neyrinck AM, Jamar F, Starkel P, Intestinal permeability, gut-bacterial dysbiosis, and behavioral markers of alcohol-dependence severity. Proc Natl Acad Sci U S A. 2014;111(42):E4485–93. 25288760 10.1073/pnas.1415174111 PMC4210345

[27] Lam YY, Ha CW, Campbell CR, Mitchell AJ, Dinudom A, Oscarsson J, Increased gut permeability and microbiota change associate with mesenteric fat inflammation and metabolic dysfunction in diet-induced obese mice. PLoS One. 2012;7(3):e34233. 22457829 10.1371/journal.pone.0034233 PMC3311621

[28] Finegold SM, Song Y, Liu C, Hecht DW, Summanen P, Kononen E, Clostridium clostridioforme: a mixture of three clinically important species. Eur J Clin Microbiol Infect Dis. 2005;24(5):319–24. 15891914 10.1007/s10096-005-1334-6

[29] Ghose C Clostridium difficile infection in the twenty-first century. Emerg Microbes Infect. 2013;2(9):e62. 26038491 10.1038/emi.2013.62 PMC3820989

[30] Nakanishi Y, Sato T, Ohteki T. Commensal Gram-positive bacteria initiates colitis by inducing monocyte/macrophage mobilization. Mucosal Immunol. 2015;8(1):152–60. 24938744 10.1038/mi.2014.53

[31] Vacca M, Celano G, Calabrese FM, Portincasa P, Gobbetti M, De Angelis M. The Controversial Role of Human Gut Lachnospiraceae. Microorganisms. 2020;8(4). 10.3390/microorganisms8040573 PMC7232163 32326636

[32] Jandhyala SM, Talukdar R, Subramanyam C, Vuyyuru H, Sasikala M, Nageshwar Reddy D. Role of the normal gut microbiota. World J Gastroenterol. 2015;21(29):8787–803. 26269668 10.3748/wjg.v21.i29.8787 PMC4528021

[33] Desai MS, Seekatz AM, Koropatkin NM, Kamada N, Hickey CA, Wolter M, A Dietary Fiber-Deprived Gut Microbiota Degrades the Colonic Mucus Barrier and Enhances Pathogen Susceptibility. Cell. 2016;167(5):1339–53 e21. 27863247 10.1016/j.cell.2016.10.043 PMC5131798

[34] Treangen TJ, Wagner J, Burns MP, Villapol S. Traumatic Brain Injury in Mice Induces Acute Bacterial Dysbiosis Within the Fecal Microbiome. Front Immunol. 2018;9:2757. 30546361 10.3389/fimmu.2018.02757 PMC6278748

[35] Nicholson SE, Watts LT, Burmeister DM, Merrill D, Scroggins S, Zou Y, Moderate Traumatic Brain Injury Alters the Gastrointestinal Microbiome in a Time-Dependent Manner. Shock. 2019;52(2):240–8. 29953417 10.1097/SHK.0000000000001211

[36] Aghakhani N Relationship between mild traumatic brain injury and the gut microbiome: A scoping review. J Neurosci Res. 2021. 10.1002/jnr.25004 34964504

[37] Derrien M, Belzer C, de Vos WM. Akkermansia muciniphila and its role in regulating host functions. Microb Pathog. 2017;106:171–81. 26875998 10.1016/j.micpath.2016.02.005

[38] Wang H, Zhang S, Yang F, Xin R, Wang S, Cui D, The gut microbiota confers protection in the CNS against neurodegeneration induced by manganism. Biomed Pharmacother. 2020;127:110150. 32330797 10.1016/j.biopha.2020.110150

[39] Pridmore RD, Berger B, Desiere F, Vilanova D, Barretto C, Pittet AC, The genome sequence of the probiotic intestinal bacterium Lactobacillus johnsonii NCC 533. Proc Natl Acad Sci U S A. 2004;101(8):2512–7. 14983040 10.1073/pnas.0307327101 PMC356981

[40] Wilson KH. The microecology of Clostridium difficile. Clin Infect Dis. 1993;16 Suppl 4:S214–8. 8324122 10.1093/clinids/16.supplement_4.s214

[41] Rolfe RD. Role of volatile fatty acids in colonization resistance to Clostridium difficile. Infect Immun. 1984;45(1):185–91. 6735467 10.1128/iai.45.1.185-191.1984 PMC263298

[42] Agarwal N, Port JD. Neuroimaging: Anatomy Meets Function: Springer; 2018.

[43] Dahmani L, Courcot B, Near J, Patel R, Amaral RSC, Chakravarty MM, Fimbria-Fornix Volume Is Associated With Spatial Memory and Olfactory Identification in Humans. Front Syst Neurosci. 2019;13:87. 32009912 10.3389/fnsys.2019.00087 PMC6971190

[44] Yin B, Li DD, Huang H, Gu CH, Bai GH, Hu LX, Longitudinal Changes in Diffusion Tensor Imaging Following Mild Traumatic Brain Injury and Correlation With Outcome. Front Neural Circuits. 2019;13:28. 31133818 10.3389/fncir.2019.00028 PMC6514143

[45] Yu J, Zhu H, Taheri S, Monday WL, Perry S, Kindy MS. Reduced Neuroinflammation and Improved Functional Recovery after Traumatic Brain Injury by Prophylactic Diet Supplementation in Mice. Nutrients. 2019;11(2). 10.3390/nu11020299 PMC6412510 30708954

[46] Ma D, Wang AC, Parikh I, Green SJ, Hoffman JD, Chlipala G, Ketogenic diet enhances neurovascular function with altered gut microbiome in young healthy mice. Sci Rep. 2018;8(1):6670. 29703936 10.1038/s41598-018-25190-5 PMC5923270

[47] Moore R, Pothoulakis C, LaMont JT, Carlson S, Madara JL. C. difficile toxin A increases intestinal permeability and induces Cl-secretion. Am J Physiol. 1990;259(2 Pt 1):G165–72. 2116728 10.1152/ajpgi.1990.259.2.G165

[48] Vekaria HJ, Talley Watts L, Lin AL, Sullivan PG. Targeting mitochondrial dysfunction in CNS injury using Methylene Blue; still a magic bullet? Neurochem Int. 2017;109:117–25. 28396091 10.1016/j.neuint.2017.04.004 PMC5632129

[49] Hammond TC, Xing X, Wang C, Ma D, Nho K, Crane PK, beta-amyloid and tau drive early Alzheimer’s disease decline while glucose hypometabolism drives late decline. Commun Biol. 2020;3(1):352. 32632135 10.1038/s42003-020-1079-x PMC7338410

[50] Lin AL, Parikh I, Yanckello LM, White RS, Hartz AMS, Taylor CE, APOE genotype-dependent pharmacogenetic responses to rapamycin for preventing Alzheimer’s disease. Neurobiol Dis. 2020;139:104834. 32173556 10.1016/j.nbd.2020.104834 PMC7486698

[51] Lin AL, Jahrling JB, Zhang W, DeRosa N, Bakshi V, Romero P, Rapamycin rescues vascular, metabolic and learning deficits in apolipoprotein E4 transgenic mice with pre-symptomatic Alzheimer’s disease. J Cereb Blood Flow Metab. 2017;37(1):217–26. 26721390 10.1177/0271678X15621575 PMC5167110

[52] Hammond TC, Lin AL. Glucose Metabolism is a Better Marker for Predicting Clinical Alzheimer’s Disease than Amyloid or Tau. J Cell Immunol. 2022;4(1):15–8. 35373192 PMC8975178

[53] Hammond TC, Xing X, Yanckello LM, Stromberg A, Chang YH, Nelson PT, Human Gray and White Matter Metabolomics to Differentiate APOE and Stage Dependent Changes in Alzheimer’s Disease. J Cell Immunol. 2021;3(6):397–412. 35265943 10.33696/immunology.3.123 PMC8903196

[54] Van Skike CE, Lin AL, Roberts Burbank R, Halloran JJ, Hernandez SF, Cuvillier J, mTOR drives cerebrovascular, synaptic, and cognitive dysfunction in normative aging. Aging Cell. 2020;19(1):e13057. 31693798 10.1111/acel.13057 PMC6974719

[55] Lin AL, Butterfield DA, Richardson A. mTOR: Alzheimer’s disease prevention for APOE4 carriers. Oncotarget. 2016;7(29):44873–4. 27385004 10.18632/oncotarget.10349 PMC5216689

[56] Lin AL, Powell D, Caban-Holt A, Jicha G, Robertson W, Gold BT, (1)H-MRS metabolites in adults with Down syndrome: Effects of dementia. Neuroimage Clin. 2016;11:728–35. 27330972 10.1016/j.nicl.2016.06.001 PMC4908308

[57] Lin AL, Fox PT, Hardies J, Duong TQ, Gao JH. Nonlinear coupling between cerebral blood flow, oxygen consumption, and ATP production in human visual cortex. Proc Natl Acad Sci U S A. 2010;107(18):8446–51. 20404151 10.1073/pnas.0909711107 PMC2889577

[58] Lin AL, Fox PT, Yang Y, Lu H, Tan LH, Gao JH. Evaluation of MRI models in the measurement of CMRO2 and its relationship with CBF. Magn Reson Med. 2008;60(2):380–9. 18666102 10.1002/mrm.21655 PMC2612533

[59] Uh J, Lin AL, Lee K, Liu P, Fox P, Lu H. Validation of VASO cerebral blood volume measurement with positron emission tomography. Magn Reson Med. 2011;65(3):744–9. 21337407 10.1002/mrm.22667 PMC3059074

[60] Kiraly M, Kiraly SJ. Traumatic brain injury and delayed sequelae: a review--traumatic brain injury and mild traumatic brain injury (concussion) are precursors to later-onset brain disorders, including early-onset dementia. ScientificWorldJournal. 2007;7:1768–76. 18040539 10.1100/tsw.2007.269 PMC5901335

